# Crystal Structure of Yeast DNA Polymerase ε Catalytic Domain

**DOI:** 10.1371/journal.pone.0094835

**Published:** 2014-04-14

**Authors:** Rinku Jain, Kanagalaghatta R. Rajashankar, Angeliki Buku, Robert E. Johnson, Louise Prakash, Satya Prakash, Aneel K. Aggarwal

**Affiliations:** 1 Department of Structural and Chemical Biology, Mount Sinai School of Medicine, New York, New York, United States of America; 2 Department of Chemistry and Chemical Biology, Cornell University and NE-CAT, Advanced Photon Source, Argonne, Illinois, United States of America; 3 Department of Biochemistry and Molecular Biology, University of Texas Medical Branch, Galveston, Texas, United States of America; New England Biolabs, Inc., United States of America

## Abstract

DNA polymerase ε (Polε) is a multi-subunit polymerase that contributes to genomic stability via its roles in leading strand replication and the repair of damaged DNA. Here we report the ternary structure of the Polε catalytic subunit (Pol2) bound to a nascent G:C base pair (Pol2_G:C_). Pol2_G:C_ has a typical B-family polymerase fold and embraces the template-primer duplex with the palm, fingers, thumb and exonuclease domains. The overall arrangement of domains is similar to the structure of Pol2_T:A_ reported recently, but there are notable differences in their polymerase and exonuclease active sites. In particular, we observe Ca^2+^ ions at both positions A and B in the polymerase active site and also observe a Ca^2+^ at position B of the exonuclease site. We find that the contacts to the nascent G:C base pair in the Pol2_G:C_ structure are maintained in the Pol2_T:A_ structure and reflect the comparable fidelity of Pol2 for nascent purine-pyrimidine and pyrimidine-purine base pairs. We note that unlike that of Pol3, the shape of the nascent base pair binding pocket in Pol2 is modulated from the major grove side by the presence of Tyr431. Together with Pol2_T:A_, our results provide a framework for understanding the structural basis of high fidelity DNA synthesis by Pol2.

## Introduction

The bulk of DNA synthesis in eukaryotes is carried out by three polymerases: Pols α, δ, and ε [1], [2]. Polα primes the Okazaki fragments on the lagging strand, which are then elongated by Polδ. Polε is believed to be the leading strand polymerase and, like Polδ, achieves fidelity via both accurate DNA polymerization and 3′→5′ proofreading exonuclease (Exo) activities. The DNA polymerization (Pol) activities of Pols δ and ε achieve an error rate of ∼10^−5^, which is then further lowered to ∼10^−7^ by their proofreading functions. DNA mismatch repair achieves another ∼100-fold increase in fidelity, for an error rate of ∼10^−9^ following DNA synthesis. Accurate DNA replication by Pols δ and ε is thus crucial in maintaining genome integrity and mutations that lower the fidelity of these polymerases lead to tumor development. Germline and somatic mutations in the exonuclease domains of Pols δ and ε are frequently associated with endometrial and colon cancers [3], [4], [5], [6].

Pols α, δ, and ε belong to the B-family of DNA polymerases. Crystal structures of the Polα [7] and Polδ [8] catalytic subunits (Pol1 and Pol3, respectively) have been determined and reveal a characteristic B-family polymerase fold comprised of a palm domain that carries the catalytic residues for dNTP addition, a fingers domain that drapes over the nascent base pair, a thumb domain that makes contacts in the DNA minor groove, and an N-terminal domain (NTD). Pol3 also contains an active exonuclease domain, whereas in Pol1 the exonuclease domain is rendered inactive due to mutations. The ternary structure of the yeast Polε catalytic domain (Pol2) was reported recently by Johansson and colleagues [9]. The structure with a nascent T:A base pair (Pol2_T:A_) was solved by molecular replacement (MR) using the Pol3 structure as a search model. We present here a crystal structure of yeast Pol2 catalytic domain that differs from the Pol2_T:A_ structure in containing a slightly different protein construct (residues 1–1187 versus 1–1228), a different template-primer, a different incoming nucleotide (dCTP versus dATP), a different nascent base pair (G:C versus T:A), and different metals (Ca^2+^ versus Mg^2+^). Also, the protein construct we used contains wild-type residues in the exonuclease domain, as opposed to mutations in the Pol2_T:A_ structure (D290A/E292A) which renders it exonuclease deficient. We show here that the two structures are very similar in their overall arrangement but differ in the Pol and Exo active sites. Pol2 is the only eukaryotic B-family polymerase for which structures are now available with both G:C and T:A nascent base pairs. Together with the Pol2_T:A_ structure, our results provide structural insights into the high fidelity of Pol2 for nascent Watson-Crick base pairs.

## Results

### Structure determination

We crystallized the Pol2 catalytic core (residues 1–1187) in ternary complex with a 12-nt/16-nt primer/template presenting G as the templating base, and with dCTP as the incoming nucleotide. To prevent degradation of the DNA by the Pol2 exonuclease activity, we prepared the protein-DNA complex in the presence of Ca^2+^ (rather than Mg^2+^). The cocrystals diffract to 2.8 Å resolution with synchrotron radiation (Argonne National Laboratory) and belong to space group C2 with unit cell dimensions of a = 147.29 Å, b = 68.48 Å, c = 149.08 Å, and β = 109.6° (Table 1). The space group is the same as that of Pol2_T:A_ cocrystals and the unit cell dimensions are very similar. Our attempts to determine the structure by molecular replacement (MR) methods using the Pol3 structure as a search model resulted in a satisfactory MR solution. However, the structure could not be refined to produce a final model. Johansson and colleagues were successful in solving the Pol2_T:A_ structure by MR using the Pol3 structure, possibly because the X-ray data extended to higher resolution (2.2 Å). Using the Pol2_T:A_ structure, we obtained an MR solution for the Pol2_G:C_ complex that readily refined to satisfactory agreement factors. The refined model of Pol2_G:C_ consists of residues 1–1187 (with missing segments 1–29, 215–219, 225–231, 665–677) of Pol2, nucleotides 1–15 of the template, nucleotides 2–12 of the primer, incoming dCTP, 4 Ca^2+^ ions, 1 Na^+^ ion, 147 solvent molecules, and 1 molecule of ethylene glycol.

**Table 1:** Data collection and refinement statistics.

**Data collection statistics**	
Space group	C2
Unit Cell dimensions	
a, b, c (Å)	147.29, 68.48, 149.08
β (°)	109.6
Resolution (Å)	50–2.8 (2.85–2.8)
R_symm_	6.3 (34.0)
I/σ (I)	25.8 (3.5)
Completeness (%)	98.9 (83.9)
Total number of reflections	127525
Number of unique reflections	34036
**Refinement statistics**	
Resolution (Å)	46.8–2.8
Number of reflections	34031
R_work_/R_free_ (%)	18.3/24.7
Number of atoms	
Protein	8851
DNA	531
Incoming dCTP	28
Ca^2+^ ions/Na^2+^ ion	4/1
Water	147
Other	10
B factors (Å^2^)	
Protein	48.5
DNA	41.3
Incoming dCTP	29.0
Ca^2+^ ion/Na^2+^ ion	62.2/26.1
Water	40.8
Other	67.4
R. m. s. deviations	
Bonds (Å)	0.011
Angles (°)	1.05
Ramachandran Plot Quality	
Allowed (%)	99.2
Disallowed (%)	0.8

A single crystal was used or solving this structure. Values for outermost shells are given in parentheses

### Overall Arrangement

**Figure 1:**
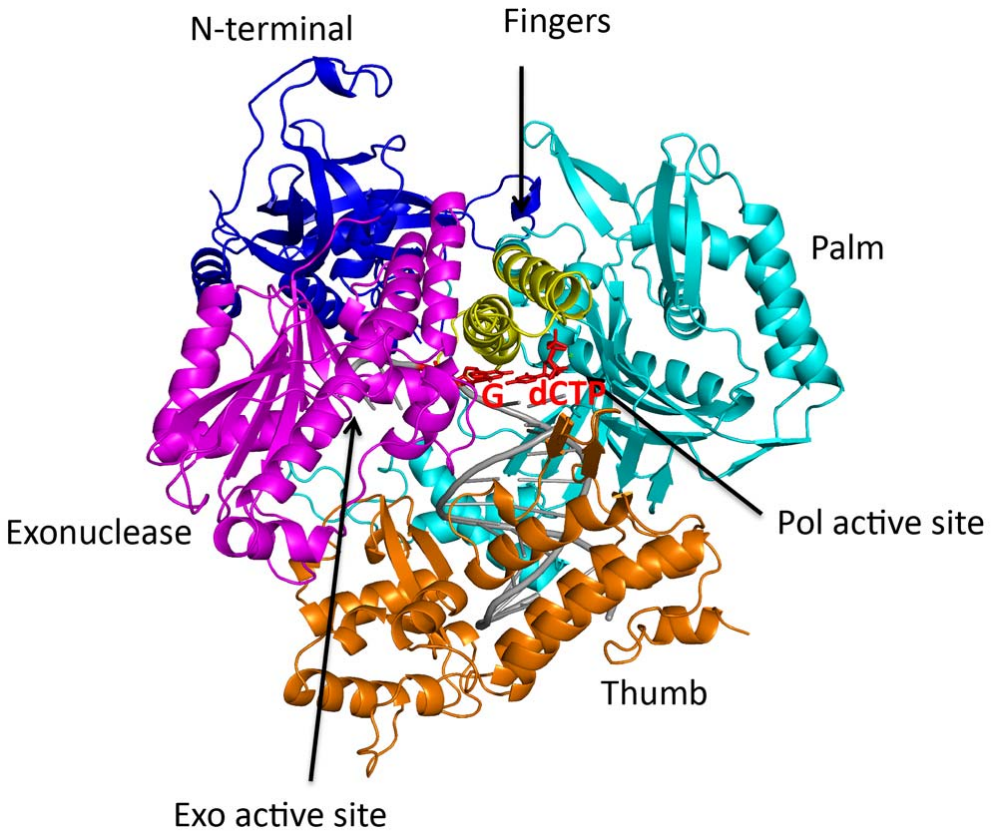
Structure of Pol2-DNA-dCTP ternary complex. Pol2 palm, fingers, thumb, exonuclease and N-terminal domains are shown in cyan, yellow, orange, magenta and blue respectively. DNA is in gray, and the templating G and incoming dCTP are in red. The polymerase (Pol) and exonuclease (Exo) active sites are labeled.

As in the Pol2_T:A_ structure, the Pol2_G:C_ catalytic core surrounds the template-primer with the palm, fingers, thumb and exonuclease domains (Figure 1). The palm interacts with the replicative end of the DNA and carries the active site residues (Asp640 and Asp877) for DNA synthesis. The fingers domain is composed of two long anti-parallel α-helices that drape over the nascent G:C base pair (Figure 1). These α-helices are longer by about two turns when compared to the helices in fingers domains of Pol1 [7] and Pol3 [8]. The thumb domain interacts primarily with the duplex portion of the template-primer and can be further divided into two subdomains that pack against the palm and exonuclease domains, respectively (Figure 1). The exonuclease domain lies on the opposite side of the DNA as the thumb domain and contains the catalytic residues (Asp290, Glu292 and Asp477) for proofreading activity. The NTD bridges the exonuclease and fingers domains. The role of the NTD in Pol2 function is unclear at present; in Pol3, the NTD has been suggested to bind both RNA and DNA [8]. The unpaired segment of the DNA template strand kinks sharply out of the Pol2 polymerase active site cleft and tracks a path between the fingers and exonuclease domains. The duplex portion of the template-primer has a B-DNA like conformation with average helical twist and rise values of 34.2 ° and 3.2 Å, respectively. Overall, the Pol2_T:A_ and Pol2_G:C_ structures are very similar with the enzymes superimposing with an rmsd of ∼0.38 Å (for 1001 Cαs) (Figure S1).

### An Extended Palm Domain

**Figure 2:**
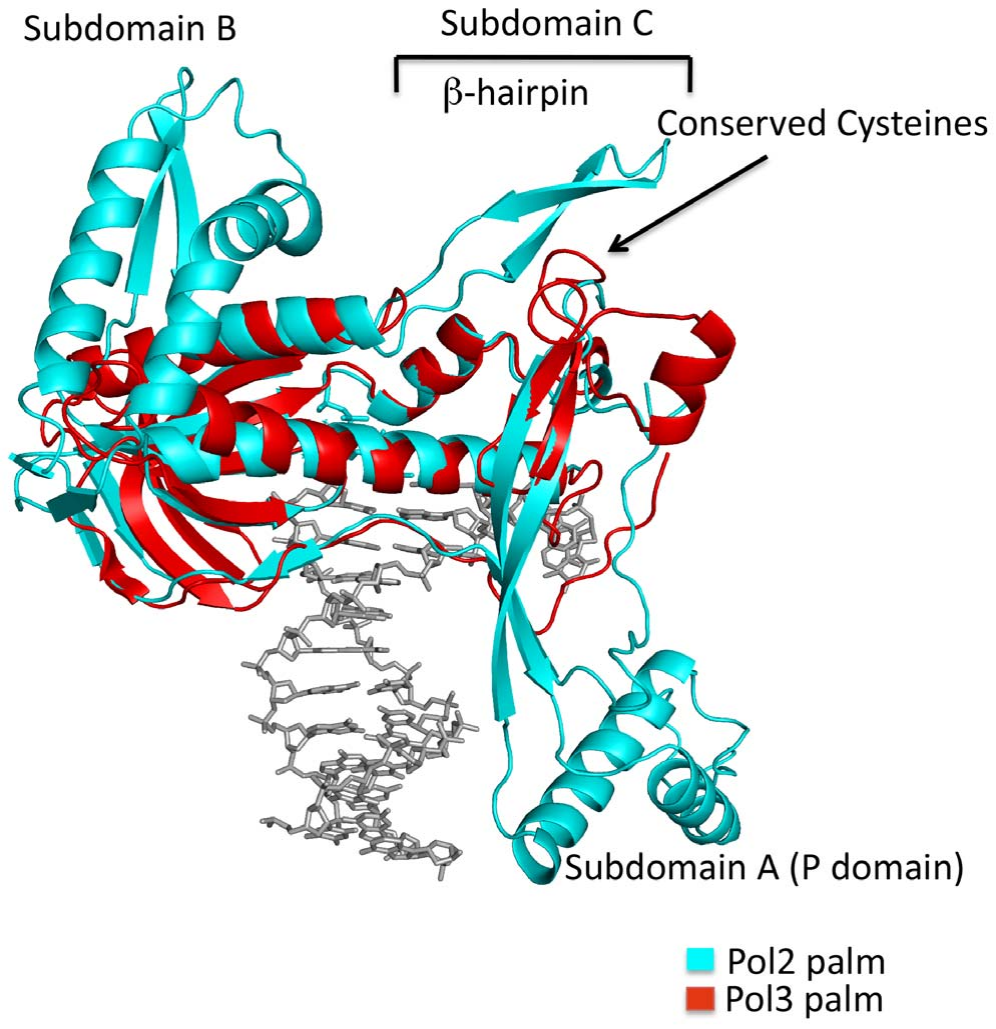
Comparison between the palm domains of Pol2 and Pol3. Pol2 palm domain is in cyan and the Pol3 palm domain is in red. DNA is in gray. Structural insertions in the Pol2 palm domain are highlighted. The Pol2 palm domain is more elaborate and contains insertions that form three subdomains – A, B, and C.

The palm domain is larger and more elaborate than that in Pol1 and Pol3 (Figure 2). In particular, the palm domain contains several insertions that coalesce to define three new subdomains (A, B and C) that extend outward from the α/β core (Figure 2). Subdomain A is delineated by residues 533–555 and 682–760 and has been named the P domain by Johansson and colleagues [9]. The P domain (or subdomain A) consists of a three-stranded β-sheet capped by two α-helices which extends towards the thumb domain. Residues Arg686, Arg744, Arg749 and Lys751 emanate from the β-sheet and are in the proximity of the duplex portion of the template-primer, in the same manner as in the Pol2_T:A_ structure. These amino acids line the major groove side of the DNA and have been suggested as a basis for the higher processivity of Pol2 as compared to Pol3 [9]. Subdomain B is delineated by residues 569–634 and 886–904 and extends towards the “extra” turns in the Pol2 fingers domain (Figure 2). Interestingly, the presence of subdomain B and the extra helical turns in the fingers domain make the polymerase active site less solvent accessible than that in Pol1 and Pol3 and may lead to a slower rate of dNTP binding or β,γ-pyrophosphate release. Subdomain C is delineated by residues 665–677 that define a putative metal binding motif and residues 919–939 that fold into a β-hairpin (Figure 2). The metal binding motif contains cysteines (Cys665, Cys668, Cys677 and Cys763) that are conserved in Pol2 orthologs but not in other B-family polymerases [10]. Surprisingly, this motif is partially disordered in both the Pol2_G:C_ and Pol2_T:A_ structures.

### Basis of High Fidelity

**Figure 3:**
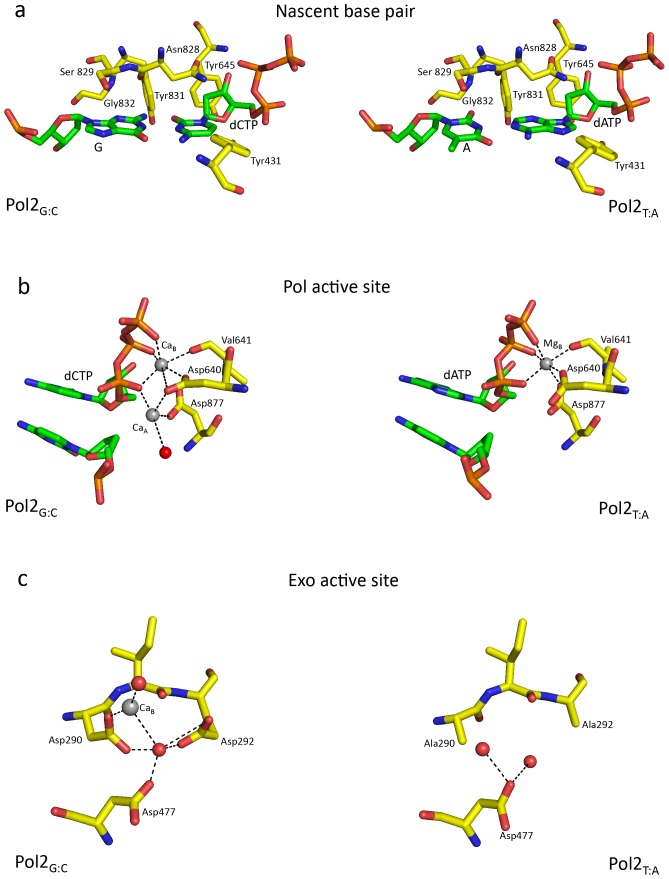
Comparison between the Pol2_G:C_ and Pol2_T:A_ polymerase and exonuclease active sites. (a) The nascent base pair binding pocket is shaped by residues Val825 (not shown for clarity), Asn828, Ser829, Ty831 and Gly832 from the fingers domain, and by Tyr645 from the palm domain. Tyr431 approaches the incoming nucleotide from the major groove side. Contacts to the G:C and T:A base pairs are interchangeable in the two structures. (b) The polymerase active site is characterized by acidic residues Asp640 and Asp877. The Pol2_G:C_ (this work) structure has two Ca^2+^ ions (gray spheres) at positions A and B in the polymerase active site. Pol2_T:A_ structure was crystallized with one Mg^2+^ ion in the active site. (c) Exonuclease active site in Pol2_GC_ (left) and Pol2_TA_ (right). Ca^2+^ ion at position B of Pol2_G:C_ is shown as gray sphere and is coordinated by Asp290, and a water molecule (red sphere). The atom at position A was modeled as water due to its close proximity to the metal ion at position B. In the Pol2_T:A_ structure, the exonuclease catalytic residues (Asp290 and Glu292) were mutated to alanines and there are no bound metal ions.

Pol2's fidelity for a nascent Watson-Crick (W-C) base pair is determined primarily by residues Val825, Asn828, Ser829, Ty831 and Gly832 from the fingers domain, and by Tyr645 from the palm domain (Figure 3a). Most of the contacts to the nascent G:C base pair occur from atop (Asn828 and Ser829) or from the minor groove side (Tyr 645, Tyr831, and Gly832). These contacts are primarily van der Waals (vdW) in nature and are also maintained in the Pol2_T:A_ structure. For example, the vdW contacts to the minor groove acceptors N3 and O2 of the nascent G:C base pair in the Pol2_G:C_ structure are simply switched to O2 and N3 atoms of the T:A base pair Pol2_T:A_ structure (Figure 3a). Conservation of base-pair interactions in the Pol2_T:A_ and Pol2_G:C_ structure reflects the accuracy of Pol2 for pyrimidine-purine and purine-pyrimidine base-pairs.

Unique to Pol2, is the presence of Tyr431 in the major groove of the nascent W-C base pair. Tyr431 lies in a loop (residues 430–439) of the exonuclease domain and approaches the incoming dCTP from the major groove side with its hydroxyl group located ∼4.0 Å from the dCTP N4 atom (Figure 3a). In the structure of Pol3, the major grove is devoid of contacts as the corresponding amino acid Lys473 is disordered and lies >7 Å away from the nascent base pair (Figure S2).

### Polymerase and Exonuclease Active sites

The Pol2 polymerase and exonuclease active site are separated by ∼41 Å in a direction roughly perpendicular to the DNA axis (Figure 1). The polymerase active site is characterized by acidic residues Asp640 and Asp877 and two calcium ions (A and B) (Figure 3b). Ca^2+^ A and B are separated by ∼3.6 Å and are analogous to metals “A” and “B” in other DNA polymerases [11], [12], [13], [14]. Ca^2+^ A is more mobile than Ca^2+^ B (B-factor of 68 Å^2^ versus 49 Å^2^). Although calcium inhibits Pol2 activity, the active site geometry is appropriate for the two-metal mechanism of catalysis [15] with the putative 3′OH located ∼3.8 Å from the dCTP α-phosphate and aligned with respect to the Pα -O3′ bond (angle of about 148°). Metals A and B are in a position to activate the primer 3′OH for its nucleophilic attack on the dNTP α-phosphate and to stabilize the pentacovalent transition state.

The exonucleolytic reaction in B-family polymerases is also believed to proceed by a two-metal mechanism [16], with metals occupying sites A and B in the exonuclease domain [17], [18], [19]. Since the exonuclease catalytic residues (Asp290 and Glu292) were mutated in the Pol2_T:A_ structure there are no bound metal ions [9]. By contrast, we observe Ca^2+^ at site B, coordinated by residue Asp290 (Figure 3c). We also observe strong electron density next to this Ca^2+^, suggestive of a second Ca^2+^ ion bound at site A (Figure S3). However, the distance between the two putative calcium ions would then be ∼3.2 Å, which is shorter than the typical distance of ∼3.7–4.0 Å. Given this uncertainty, we have assigned this density at site A as a water molecule, though refining it as such leads to substantial positive electron density in an F_o_-F_c_ map (indicative of a more electron-rich atom) (Figure S3).

## Discussion

Pol2_G:C_ and Pol2_T:A_ structures are very similar, including regions of the enzyme and DNA that are visible in the electron density map. Importantly, contacts to the nascent G:C and T:A base pairs are interchangeable and reflect the roughly equal fidelity of Pol2 for Pu:Py or Py:Pu nascent base pairs (Figure S4). A notable difference between the two structures is the number of metals in the polymerase active site. Johansson and colleagues crystallized Pol2_T:A_ in the presence of Mg^2+^; to prevent degradation of the DNA by the Pol2 exonuclease activity residues Asp290 and Glu292 were mutated to alanines. The Pol2_T:A_ structure shows a single Mg^2+^ ion in the polymerase active site at position B (Figure 3b). By contrast, we cocrystallized Pol2_G:C_ with the wild-type enzyme; to prevent exonucleolytic degradation of DNA we used Ca^2+^ in place of Mg^2+^. The structure reveals Ca^2+^ ions at positions A and B in the polymerase active site. Typically, metal A in DNA polymerases is coordinated by the α-phosphate of the incoming nucleotide, the putative primer 3′OH, the carboxylates of active site residues, and water molecules. It tends to be more mobile than metal B with longer ligation distances and is often not observed in DNA polymerases. For example, the structures of Polκ show only a single Mg^2+^ at position B [20]. Also, the structure of Polι with incoming dTTP showed a single Mg^2+^ at position B [21], but a later structure with incoming dCTP showed a second Mg^2+^ at position A [22]. The presence of Ca^2+^ at position A in the Pol2_G:C_ structure show that the Pol2 active site is fully capable of binding a second metal. The absence of metal A in the Pol2_T:A_ structure reflects its intrinsic mobility (compared to metal B) and the lack of a 3′OH ligand at the primer terminus.

A unique feature of Pol2 is the presence of Tyr431 in the major groove of the nascent base pair binding pocket (Figure 3a and S2). In both Pol2 and Pol3, the binding pocket is primarily shaped by residues from the palm and fingers domain which are conserved in all B-family polymerases [8]. The interactions of these residues with the nascent base pair occur from the top or the minor groove side and the binding pocket is devoid of interactions in the major groove. The binding pocket of Pol2 is also shaped by Tyr431 from the exonuclease domain that approaches the nascent base pair from the major groove side. Figure S2a shows the structures of Pol2 and Pol3 superimposed by their palm domains. Relative to that of Pol2, the entire exonuclease domain of Pol3 is shifted up and away from the major groove by >5 Å. This results in Lys473 of Pol3 (which is equivalent to Tyr431 of Pol2) being positioned >7 Å away from the incoming nucleotide. This may lend to differences in base substitution errors between Pol2 and Pol3 [23], [24]. A better understanding of the role of Tyr431 in the fidelity of Pol2 would require structures of wild type and mutant Pol2 with different mismatches. Interestingly, if the exonuclease domain of Pol3 were in the same relative orientation as that of Pol2 (Figure S2b), a ‘β-hairpin’ from its exonuclease domain would collide with the unpaired segment of the template strand. An analogous hairpin in RB69 and T4 Pols has been proposed to facilitate strand separation and the transition of the primer strand between the polymerase and exonuclease sites [25], [26], [27]. In Pol2, this β-hairpin is much smaller but, surprisingly, does not appear to limit the ability of Pol2 to proofread insertion errors [23], [24].

An intriguing feature of Pol2 is the putative metal binding motif in the palm domain, characterized by three conserved cysteines (Cys665, Cys677 and Cys763). Based on spectroscopic and other data, we have shown that these cysteines bind a Fe-S cluster [10]. For example, wild-type Pol2 catalytic core is found to be yellowish-brown in color, but a mutant in which Cys665, Cys677 and Cys763 are mutated is colorless. We also showed that the Cys triple mutant is deficient in DNA polymerase activity but not in the exonuclease activity. This is consistent with the location of the cysteines on palm domain, remote from the exonuclease domain. Considering its functional importance, it is surprising therefore that the cysteine-rich metal binding motif is partially disordered in both the Pol2_G:C_ and Pol2_T:A_ structures. Johansson and colleagues positioned a Zn^2+^ ion in the Pol2_T:A_ structure, coordinated to Cys667 and Cys763 and to partially disordered Cys665 and Cys668. We suspect that the disorder in Cys665 and Cys668 likely reflects the binding of sub-optimal Zn^2+^ rather than a Fe-S cluster. Fe-S clusters are labile and can be substituted by Zn^2+^ and it is quite possible that the Pol2 form that crystallizes in both Pol2_T:A_ and Pol2_G:C_ structures contains Zn^2+^ instead of a functional Fe-S cluster. It will be interesting to grow the crystals of Pol2 under anaerobic conditions to see whether a Fe-S cluster will replaces Zn^2+^ and whether this lead to the ordering of the cysteines.

## Methods

### Protein and DNA preparation

The catalytic core of *S. cerevisiae* Pol2 (residues 1–1187) harboring a N-terminal GST tag was expressed in the protease deficient yeast strain YRP654. The GST tag was engineered to be cleaved with PreScission protease. Protein was purified by affinity chromatography with Glutathione Sepharose 4B beads, removal of the GST tag by cleavage with PreScission protease, and further purification by size exclusion on a Superdex 200 column (GE Healthcare). Purified protein was concentrated and stored at −80°C until further use

The primer and template strands used for crystallization were purified by anion exchange on a MonoQ column, desalted and lyophilized before crystallization. Purified 12-nt primer harboring a dideoxycytosine at the 3′ end (ATCCTCCCCTAC^dd^) was mixed with purified 16-nt template (TAAGGTAGGGGAGGAT) in 1∶1 ratio and annealed to yield a 12/16 template-primer duplex DNA with one replicative end.

### Cocrystallization

The Pol2_G.C_ ternary complex was prepared by mixing purified Pol2 and the 12/16 template –primer DNA duplex in the ratio of 1∶1, followed by the addition of dCTP and CaCl_2_ to final concentrations of 10 mM each. The ternary complex was crystallized from solution containing 10–15% polyethylene glycol 5000 monomethyl ether and 25 mM magnesium acetate, 1% DMSO in 0.1 M Tris-HCl buffer (pH = 7.0). For data collection, crystals were cryoprotected by stepwise soaks in mother liquor solutions containing 5–25% ethylene glycol and then flash frozen in liquid nitrogen. X-ray data on cryocooled crystals were measured at Advanced Photon Source (APS, beamline 23-ID) of Argonne National Laboratory at a wavelength of 1.0332 Å. Data sets were indexed and integrated using the HKL-2000 package [28]. Crystals diffract to 2.8 Å and belong to space group C2 with unit cell dimensions of a = 147.29 Å, b = 68.48 Å, c = 149.08 Å and α = β = 90°, γ = 109.6°. Matthew's coefficient suggested one protein molecule in the asymmetric unit.

### Structure determination and refinement

The structure of Pol2_G:C_ was solved by molecular replacement (MR), using the Pol2_T.A_ complex as a search model (with the DNA, incoming nucleotide, metal ions and water molecules omitted). The program Phaser [29] gave a unique MR solution. The first round of refinement and map calculation was carried out without the DNA using the program PHENIX [30]. The electron density maps (2F_o_-F_c_ and F_o_-F_c_) showed unambiguous densities for the DNA and incoming nucleotide, which were then included in the model for subsequent refinement. Iterative rounds of refinement and water picking were performed with PHENIX and model building with program Coot [31]. The final model has good stereochemistry as shown by MolProbity [32] with >99.4% of all residues in allowed regions of the Ramachandran plot and 0.6% in the disallowed regions. Final coordinates have been submitted to the Protein Data Bank with PDB ID 4PTF. Figures were prepared using PyMol [33].

### Structural analysis

Protein structures were aligned and superimposed using MUSTANG [34] and LSQMAN [35]. Web 3DNA (w3dna.rutgers.edu) [36] was used for analysis of DNA helical parameters.

## Supporting Information

Figure S1 **Superimposition of the structures of Pol2_G:C_ (cyan) and Pol2_T:A_ (red).** Overall, the structures are very similar.
(PDF)

Figure S2 **Major groove interactions in the nascent base pair binding pocket of Pol2_G:C_ (cyan) and Pol3_G:C_ (red).** (a) Superimposition of Pol2_G:C_ and Pol3_G:C_ by their palm domains. Pol2 residues 528∶767 and 844∶989 were aligned with Pol3 amino acids 577∶660 and 713∶834. Tyr431 of the Pol2 exonuclease domain approaches the nascent base pair from the major groove side. Compared to Pol2, the Pol3 exonuclease domain is shifted away from the major grove by >5 Å. (b) Superimposition of the exonuclease domains. If the Pol3 exo domain (residues 316∶531) were in the same relative orientation as the Pol2 exo domain (residues 284∶501), a β-hairpin (labeled above) would collide with the unpaired segment of the template strand. This β-hairpin has been implicated in aiding the transition of the primer strand between the polymerase and exonuclease active sites. The Pol2 β-hairpin is much smaller and does not interact with the DNA.
(PDF)

Figure S3 **Residual F_o_-F_c_ density (green, 3σ) in the Pol2_G:C_ exonuclease active site with position A modeled as a water molecule.** This is suggestive of a more electron rich atom (possible Ca^2+^) in the vicinity of position A.
(PDF)

Figure S4 **Schematic of protein-DNA interactions.** Amino acids from Pol2 palm, fingers, thumb, exonuclease and N-terminal domains are shown in cyan, yellow, orange, magenta and blue respectively; incoming dCTP is shown in red. A distance cut-off of 3.35 Å was used for protein-DNA interactions. Residues R744, R749 and R751 from subdomain A are in the vicinity of the DNA but at a distance larger than 3.5 Å (not shown). Figure was generated with NUCPLOT (Luscombe N M, Laskowski R A, Thornton J M (1997). NUCPLOT: a program to generate schematic diagrams of protein-DNA interactions. *Nucleic Acids Res.*, **25**, 4940-4945) and modified for clarity.
(PDF)

## References

[1] Johnson A, O'Donnell M (2005) Cellular DNA replicases: components and dynamics at the replication fork. Annu Rev Biochem 74: 283–315. 15952889 10.1146/annurev.biochem.73.011303.073859

[2] Johansson E, Macneill SA (2010) The eukaryotic replicative DNA polymerases take shape. Trends Biochem Sci 35: 339–347. 20163964 10.1016/j.tibs.2010.01.004

[3] Briggs S, Tomlinson I (2013) Germline and somatic polymerase epsilon and delta mutations define a new class of hypermutated colorectal and endometrial cancers. J Pathol 230: 148–153. 23447401 10.1002/path.4185 PMC3709119

[4] Palles C, Cazier JB, Howarth KM, Domingo E, Jones AM, et al (2013) Germline mutations affecting the proofreading domains of POLE and POLD1 predispose to colorectal adenomas and carcinomas. Nat Genet 45: 136–144. 23263490 10.1038/ng.2503 PMC3785128

[5] Comprehensive molecular characterization of human colon and rectal cancer. Nature 487: 330–337. 22810696 10.1038/nature11252 PMC3401966

[6] Kandoth C, Schultz N, Cherniack AD, Akbani R, Liu Y, et al (2013) Integrated genomic characterization of endometrial carcinoma. Nature 497: 67–73. 23636398 10.1038/nature12113 PMC3704730

[7] Perera RL, Torella R, Klinge S, Kilkenny ML, Maman JD, et al (2013) Mechanism for priming DNA synthesis by yeast DNA Polymerase alpha. Elife 2: e00482. 23599895 10.7554/eLife.00482 PMC3628110

[8] Swan MK, Johnson RE, Prakash L, Prakash S, Aggarwal AK (2009) Structural basis of high-fidelity DNA synthesis by yeast DNA polymerase delta. Nat Struct Mol Biol 16: 979–986. 19718023 10.1038/nsmb.1663 PMC3055789

[9] Hogg M, Osterman P, Bylund GO, Ganai RA, Lundstrom EB, et al (2014) Structural basis for processive DNA synthesis by yeast DNA polymerase epsilon. Nat Struct Mol Biol 21: 49–55. 24292646 10.1038/nsmb.2712

[10] Jain R, Vanamee ES, Dzikovski BG, Buku A, Johnson RE, et al (2014) An Iron-Sulfur Cluster in the Polymerase Domain of Yeast DNA Polymerase epsilon. J Mol Biol 426: 301–308. 24144619 10.1016/j.jmb.2013.10.015 PMC4061903

[11] Franklin MC, Wang J, Steitz TA (2001) Structure of the replicating complex of a pol alpha family DNA polymerase. Cell 105: 657–667. 11389835 10.1016/s0092-8674(01)00367-1

[12] Doublie S, Tabor S, Long AM, Richardson CC, Ellenberger T (1998) Crystal structure of a bacteriophage T7 DNA replication complex at 2.2 A resolution [see comments]. Nature 391: 251–258. 9440688 10.1038/34593

[13] Li Y, Korolev S, Waksman G (1998) Crystal structures of open and closed forms of binary and ternary complexes of the large fragment of Thermus aquaticus DNA polymerase I: structural basis for nucleotide incorporation. Embo J 17: 7514–7525. 9857206 10.1093/emboj/17.24.7514 PMC1171095

[14] Nair DT, Johnson RE, Prakash L, Prakash S, Aggarwal AK (2005) Rev1 employs a novel mechanism of DNA synthesis using a protein template. Science 309: 2219–2222. 16195463 10.1126/science.1116336

[15] Rothwell PJ, Waksman G (2005) Structure and mechanism of DNA polymerases. Adv Protein Chem 71: 401–440. 16230118 10.1016/S0065-3233(04)71011-6

[16] Yang W (2011) Nucleases: diversity of structure, function and mechanism. Q Rev Biophys 44: 1–93. 20854710 10.1017/S0033583510000181 PMC6320257

[17] Beese LS, Steitz TA (1991) Structural basis for the 3′-5′ exonuclease activity of Escherichia coli DNA polymerase I: a two metal ion mechanism. EMBO J 10: 25–33. 1989886 10.1002/j.1460-2075.1991.tb07917.x PMC452607

[18] Freemont PS, Friedman JM, Beese LS, Sanderson MR, Steitz TA (1988) Cocrystal structure of an editing complex of Klenow fragment with DNA. Proc Natl Acad Sci U S A 85: 8924–8928. 3194400 10.1073/pnas.85.23.8924 PMC282619

[19] Shamoo Y, Steitz TA (1999) Building a replisome from interacting pieces: sliding clamp complexed to a peptide from DNA polymerase and a polymerase editing complex. Cell 99: 155–166. 10535734 10.1016/s0092-8674(00)81647-5

[20] Lone S, Townson SA, Uljon SN, Johnson RE, Brahma A, et al (2007) Human DNA polymerase kappa encircles DNA: implications for mismatch extension and lesion bypass. Mol Cell 25: 601–614. 17317631 10.1016/j.molcel.2007.01.018

[21] Nair DT, Johnson RE, Prakash S, Prakash L, Aggarwal AK (2004) Replication by human DNA polymerase-iota occurs by Hoogsteen base-pairing. Nature 430: 377–380. 15254543 10.1038/nature02692

[22] Nair DT, Johnson RE, Prakash L, Prakash S, Aggarwal AK (2005) Human DNA Polymerase iota Incorporates dCTP Opposite Template G via a G.C+ Hoogsteen Base Pair. Structure (Camb) 13: 1569–1577. 16216587 10.1016/j.str.2005.08.010

[23] Shcherbakova PV, Pavlov YI, Chilkova O, Rogozin IB, Johansson E, et al (2003) Unique error signature of the four-subunit yeast DNA polymerase epsilon. J Biol Chem 278: 43770–43780. 12882968 10.1074/jbc.M306893200

[24] Fortune JM, Pavlov YI, Welch CM, Johansson E, Burgers PM, et al (2005) Saccharomyces cerevisiae DNA polymerase delta: high fidelity for base substitutions but lower fidelity for single- and multi-base deletions. J Biol Chem 280: 29980–29987. 15964835 10.1074/jbc.M505236200

[25] Hogg M, Aller P, Konigsberg W, Wallace SS, Doublie S (2007) Structural and biochemical investigation of the role in proofreading of a beta hairpin loop found in the exonuclease domain of a replicative DNA polymerase of the B family. J Biol Chem 282: 1432–1444. 17098747 10.1074/jbc.M605675200

[26] Reha-Krantz LJ, Marquez LA, Elisseeva E, Baker RP, Bloom LB, et al (1998) The proofreading pathway of bacteriophage T4 DNA polymerase. J Biol Chem 273: 22969–22976. 9722519 10.1074/jbc.273.36.22969

[27] Stocki SA, Nonay RL, Reha-Krantz LJ (1995) Dynamics of bacteriophage T4 DNA polymerase function: identification of amino acid residues that affect switching between polymerase and 3′ —>5′ exonuclease activities. J Mol Biol 254: 15–28. 7473755 10.1006/jmbi.1995.0595

[28] Otwinowski Z, Minor W (1997) Processing of X-ray diffraction data collected in oscillation mode. Methods Enzymol 276: 307–326. 10.1016/S0076-6879(97)76066-X 27754618

[29] McCoy AJ, Grosse-Kunstleve RW, Adams PD, Winn MD, Storoni LC, et al (2007) Phaser crystallographic software. J Appl Crystallogr 40: 658–674. 19461840 10.1107/S0021889807021206 PMC2483472

[30] Adams PD, Afonine PV, Bunkoczi G, Chen VB, Davis IW, et al (2010) PHENIX: a comprehensive Python-based system for macromolecular structure solution. Acta Crystallogr D Biol Crystallogr 66: 213–221. 20124702 10.1107/S0907444909052925 PMC2815670

[31] Emsley P, Cowtan K (2004) Coot: model-building tools for molecular graphics. Acta Crystallogr D Biol Crystallogr 60: 2126–2132. 15572765 10.1107/S0907444904019158

[32] Chen VB, Arendall WB 3rd, Headd JJ, Keedy DA, Immormino RM, et al (2010) MolProbity: all-atom structure validation for macromolecular crystallography. Acta Crystallogr D Biol Crystallogr 66: 12–21. 20057044 10.1107/S0907444909042073 PMC2803126

[33] Delano WL (2002) The PyMol Molecular Graphics System. Delano Scientific LLC, San Carlos, USA.

[34] Konagurthu AS, Whisstock JC, Stuckey PJ, Lesk AM (2006) MUSTANG: a multiple structural alignment algorithm. Proteins 64: 559–574. 16736488 10.1002/prot.20921

[35] Kleywegt GJ, Jones TA (1995) Where freedom is given, liberties are taken. Structure 3: 535–540. 8590014 10.1016/s0969-2126(01)00187-3

[36] Zheng G, Lu XJ, Olson WK (2009) Web 3DNA—a web server for the analysis, reconstruction, and visualization of three-dimensional nucleic-acid structures. Nucleic Acids Res 37: W240–246. 19474339 10.1093/nar/gkp358 PMC2703980

