# Supplementary material for: Crystal Structure of Yeast DNA Polymerase ε Catalytic Domain
Source: PLoS One. 2014 Apr 14;9(4):e94835. doi: 10.1371/journal.pone.0094835 (PMC3986358; doi:10.1371/journal.pone.0094835)
Supplement: Figure S1 — Superimposition of the structures of Pol2G:C (cyan) and Pol2T:A (red). Overall, the structures are very similar. (PDF) [file pone.0094835.s001.pdf]

Figure S1

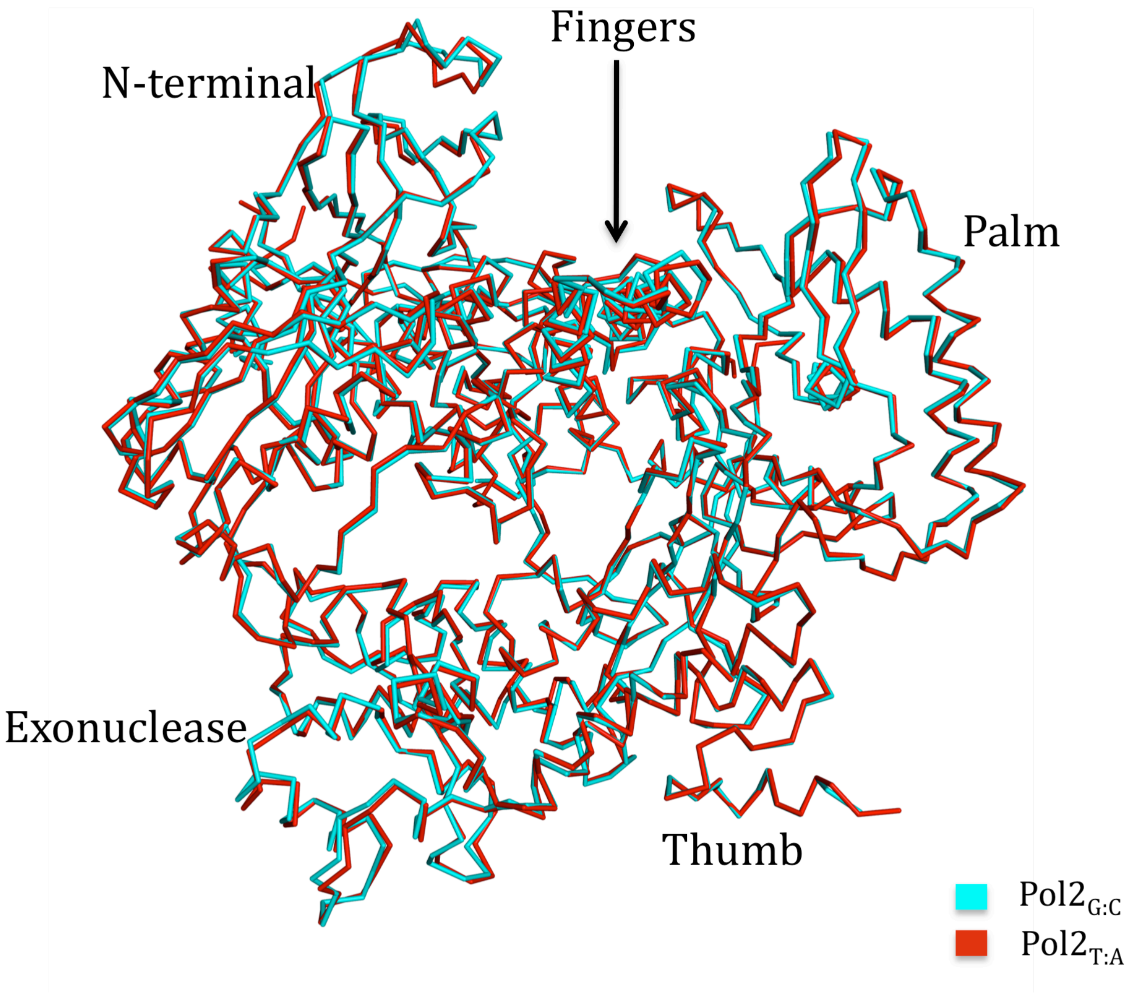

Supplementary figure 1. Superimposition of the structures of Pol2$_{G:C}$ (cyan) and Pol2$_{T:A}$ (red). Overall, the structures are very similar.
