# Supplementary material for: Crystal Structure of Yeast DNA Polymerase ε Catalytic Domain
Source: PLoS One. 2014 Apr 14;9(4):e94835. doi: 10.1371/journal.pone.0094835 (PMC3986358; doi:10.1371/journal.pone.0094835)
Supplement: Figure S2 — Major groove interactions in the nascent base pair binding pocket of Pol2G:C (cyan) and Pol3G:C (red). (a) Superimposition of Pol2G:C and Pol3G:C by their palm domains. Pol2 residues 528∶767 and 844∶989 were aligned with Pol3 amino acids 577∶660 and 713∶834. Tyr431 of the Pol2 exonuclease domain approaches the nascent base pair from the major groove side. Compared to Pol2, the Pol3 exonuclease domain is shifted away from the major grove by >5 Å. (b) Superimposition of the exonuclease domains. If the Pol3 exo domain (residues 316∶531) were in the same relative orientation as the Pol2 exo domain (residues 284∶501), a β-hairpin (labeled above) would collide with the unpaired segment of the template strand. This β-hairpin has been implicated in aiding the transition of the primer strand between the polymerase and exonuclease active sites. The Pol2 β-hairpin is much smaller and does not interact with the DNA. (PDF) [file pone.0094835.s002.pdf]

Figure S2

a. Superimposition by palm domains **b.** Superimposition by exo domains

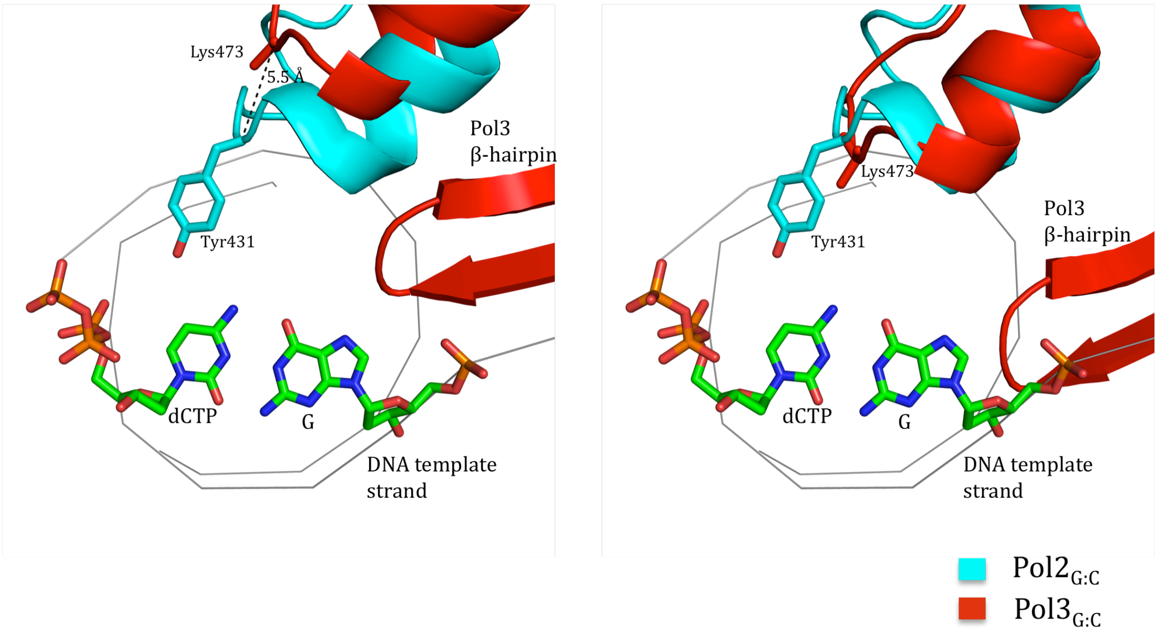

Supplementary figure 2. Major groove interactions in the nascent base pair binding pocket of $Pol2_{G:C}$ (cyan) and $Pol3_{G:C}$ (red). (a) Superimposition of $Pol2_{G:C}$ and $Pol3_{G:C}$ by their palm domains. Pol2 residues 528:767 and 844:989 were aligned with Pol3 amino acids 577:660 and 713:834. Tyr431 of the Pol2 exonuclease domain approaches the nascent base pair from the major groove side. Compared to Pol2, the Pol3 exonuclease domain is shifted away from the major grove by > 5 Å. (b) Superimposition of the exonuclease domains. If the Pol3 exo domain (residues 316:531) were in the same relative orientation as the Pol2 exo domain (residues 284:501), a β-hairpin (labeled above) would collide with the unpaired segment of the template strand. This β-hairpin has been implicated in aiding the transition of

the primer strand between the polymerase and exonuclease active sites. The Pol2 β-hairpin is much smaller and does not interact with the DNA.
