# Supplementary material for: Crystal Structure of Yeast DNA Polymerase ε Catalytic Domain
Source: PLoS One. 2014 Apr 14;9(4):e94835. doi: 10.1371/journal.pone.0094835 (PMC3986358; doi:10.1371/journal.pone.0094835)
Supplement: Figure S3 — Residual Fo-Fc density (green, 3σ) in the Pol2G:C exonuclease active site with position A modeled as a water molecule. This is suggestive of a more electron rich atom (possible Ca2+) in the vicinity of position A. (PDF) [file pone.0094835.s003.pdf]

Figure S3

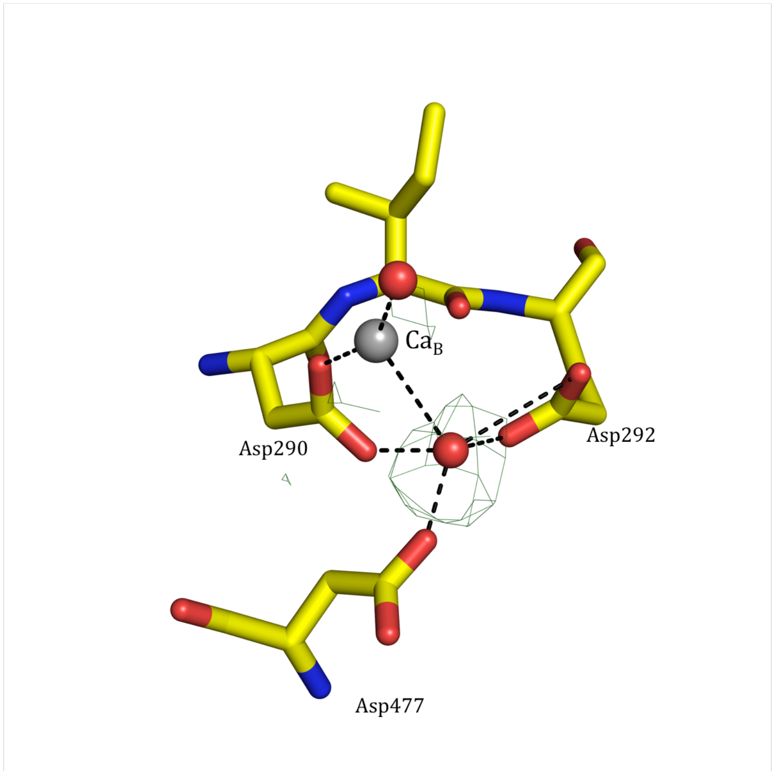

Supplementary figure 3. Residual F_o-F_c density (green, 3σ) in the $Pol2_{G:C}$ exonuclease active site with position A modeled as a water molecule. This is suggestive of a more electron rich atom (possible Ca^{2+}) in the vicinity of position A.
