# Supplementary material for: Crystal Structure of Yeast DNA Polymerase ε Catalytic Domain
Source: PLoS One. 2014 Apr 14;9(4):e94835. doi: 10.1371/journal.pone.0094835 (PMC3986358; doi:10.1371/journal.pone.0094835)
Supplement: Figure S4 — Schematic of protein-DNA interactions. Amino acids from Pol2 palm, fingers, thumb, exonuclease and N-terminal domains are shown in cyan, yellow, orange, magenta and blue respectively; incoming dCTP is shown in red. A distance cut-off of 3.35 Å was used for protein-DNA interactions. Residues R744, R749 and R751 from subdomain A are in the vicinity of the DNA but at a distance larger than 3.5 Å (not shown). Figure was generated with NUCPLOT (Luscombe N M, Laskowski R A, Thornton J M (1997). NUCPLOT: a program to generate schematic diagrams of protein-DNA interactions. Nucleic Acids Res., 25, 4940-4945) and modified for clarity. (PDF) [file pone.0094835.s004.pdf]

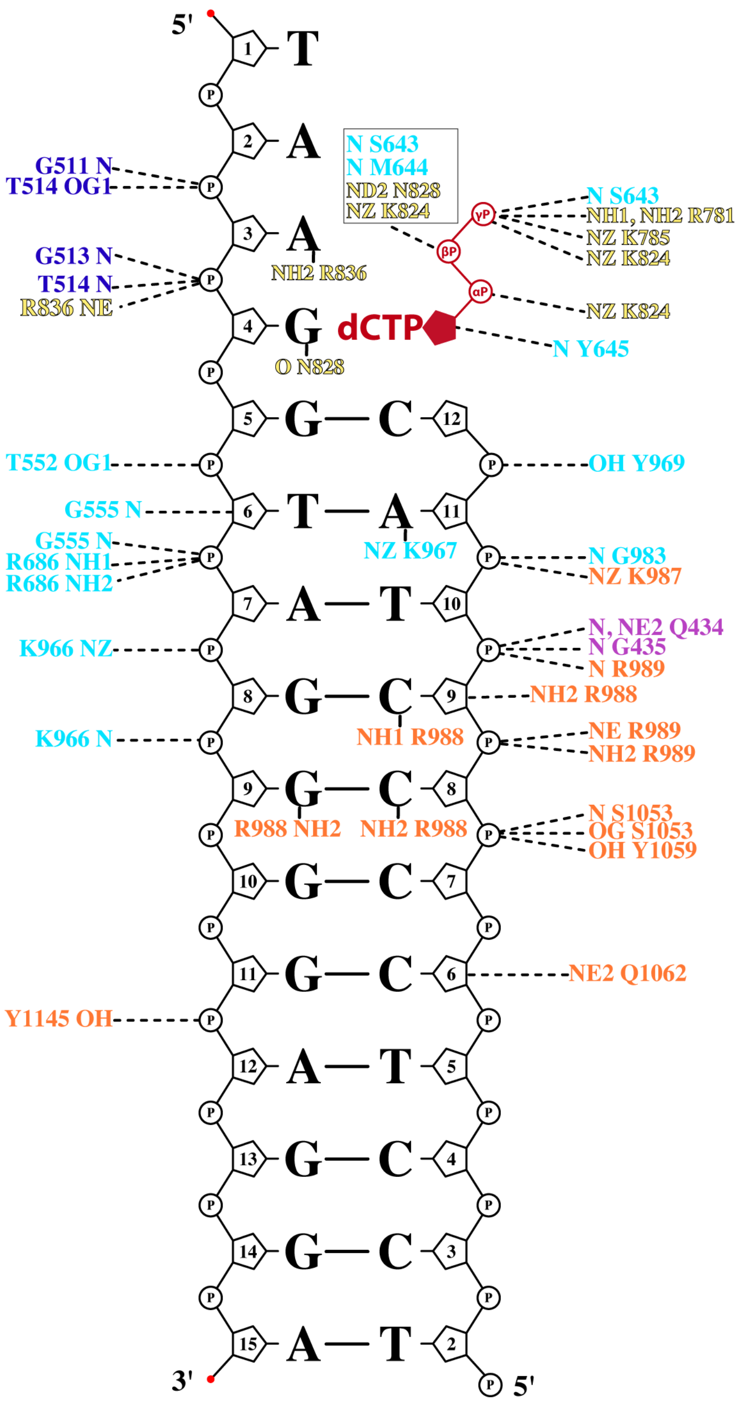

Supplementary figure 4. Schematic of protein-DNA interactions. Amino acids from Pol2 palm, fingers, thumb, exonuclease and N-terminal domains are shown in cyan, yellow, orange, magenta and blue respectively; incoming dCTP is shown in red. A

distance cut-off of 3.35 Å was used for protein-DNA interactions. Residues R744, R749 and R751 from subdomain A are in the vicinity of the DNA but at a distance larger than 3.5 Å (not shown). Figure was generated with NUCPLOT (Luscombe N M, Laskowski R A, Thornton J M (1997). NUCPLOT: a program to generate schematic diagrams of protein-DNA interactions. *Nucleic Acids Res.*, **25**, 4940-4945) and modified for clarity.
